# Supplementary material for: Distinct immune microenvironment profiles of therapeutic responders emerge in combined TGFβ/PD-L1 blockade-treated squamous cell carcinoma
Source: Commun Biol. 2021 Aug 25;4:1005. doi: 10.1038/s42003-021-02522-2 (PMC8387430; doi:10.1038/s42003-021-02522-2)
Supplement: Supplementary file 2 — Supplementary Information [file 42003_2021_2522_MOESM2_ESM.pdf]

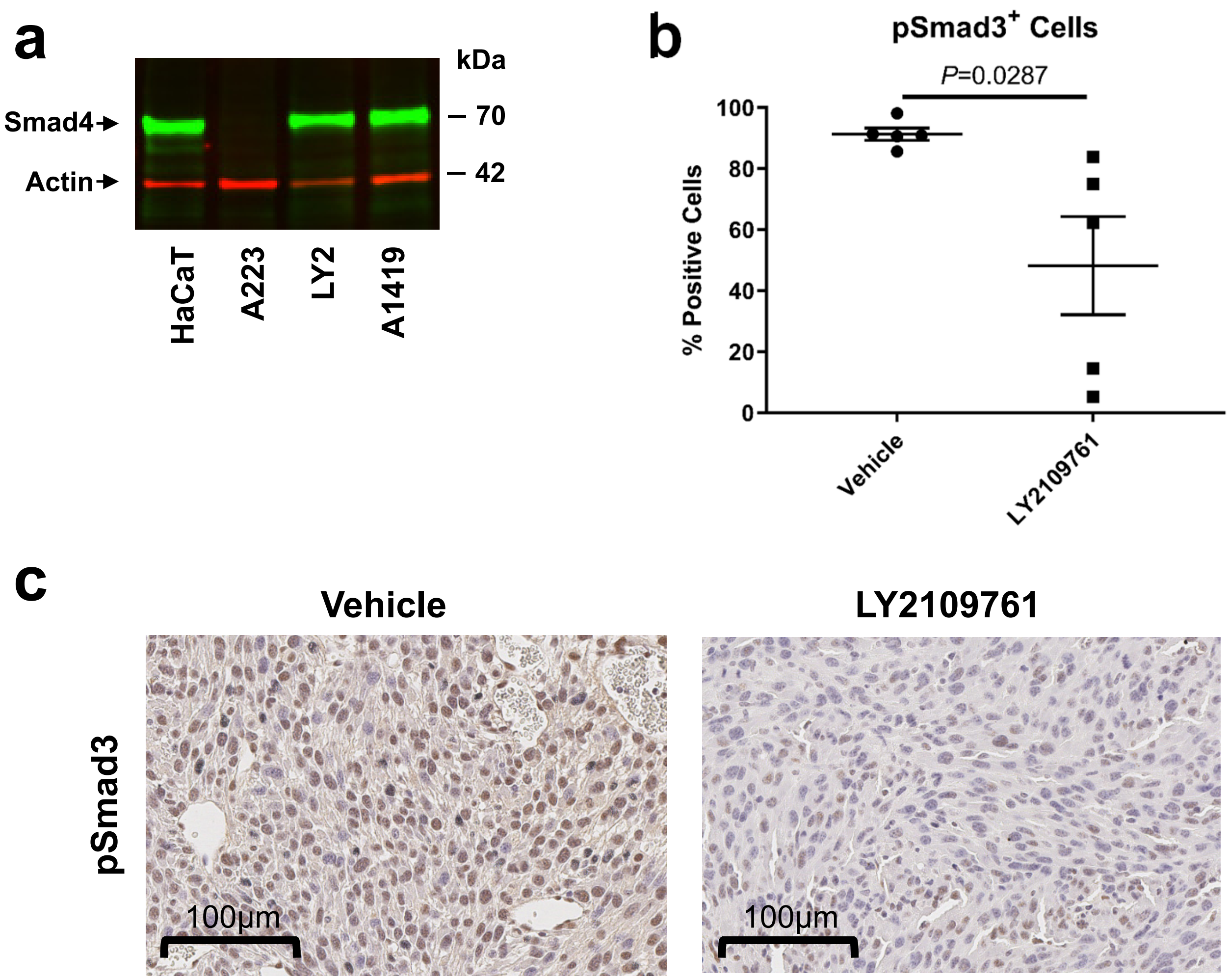

Supplementary Figure 1: LY2 PD-L1 expression requires Smad4, and human HNSCC PD-L1 expression is primarily on stromal cells.

a Smad4 protein (green, 70kDa), and actin protein (red, 42kDa) in HaCaT keratinocytes and A223, LY2, and A1419 tumor cells after western blot analysis. **b** Quantification of phopho-Smad3 (pSmad3) positive cells in A223 tumors 12 hours after treatment with 75mg/kg LY2109761 (*n*=5) or its vehicle control (*n*=5). **c** Representative pSmad3 IHC images of vehicle- or LY2109761-treated tumors quantified in **b**. An unpaired two-tailed t-test was performed for **b**, , and all error bars represent the SEM.

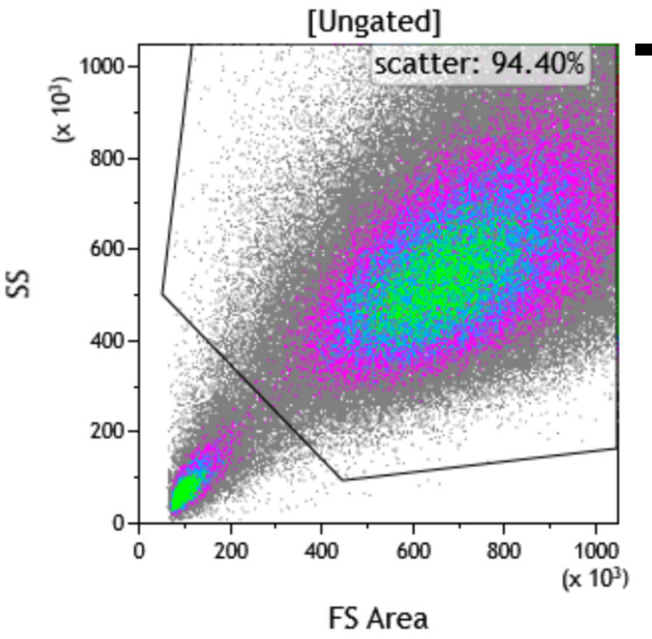

Gate	Number	%Total	%Gated
All	321,404	100.00	100.00
scatter	303,393	94.40	94.40

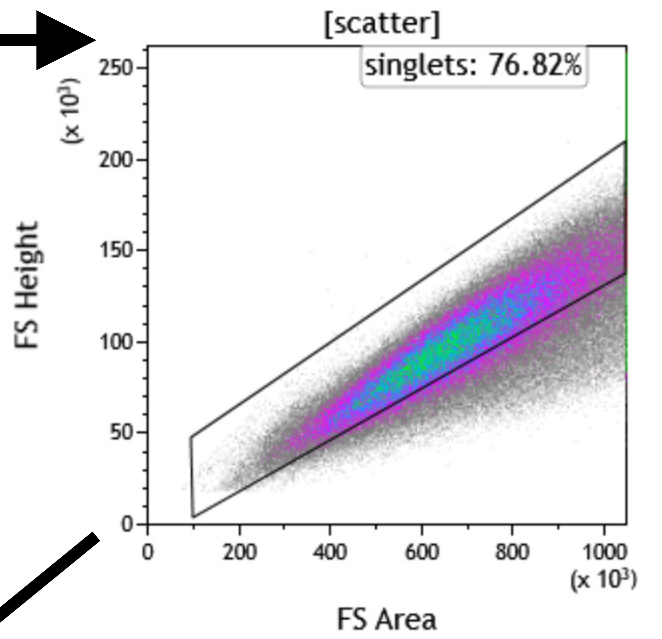

Gate	Number	%Total	%Gated
All	303,393	94.40	100.00
singlets	233,072	72.52	76.82

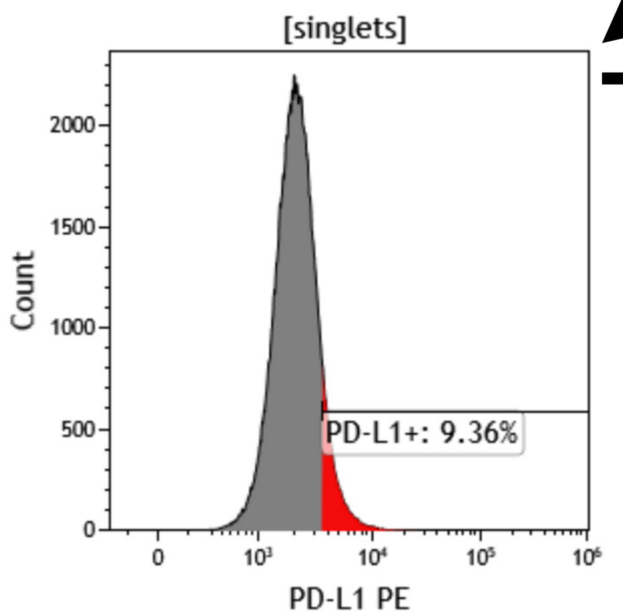

Gate	Number	%Total	%Gated	X-Med
All	233,072	72.52	100.00	2,095.51
PD-L1+	21,806	6.78	9.36	4,291.65

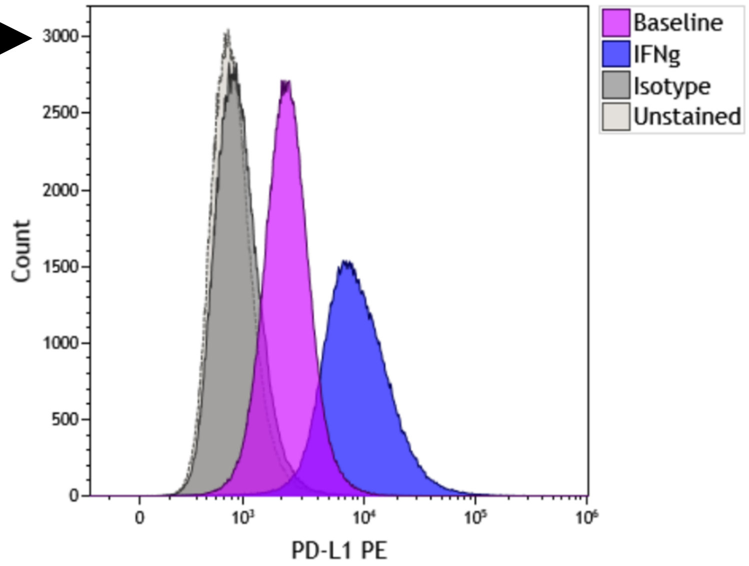

Supplementary Figure 2: Gating strategy for Fig. 2a and Supplementary Fig. 2b.

Non-debris cells gated from side scatter and forward scatter area, singlets gated by forward scatter height and area, and PD-L1$^+$ cells gating based on an isotype control compared to unstained cells.

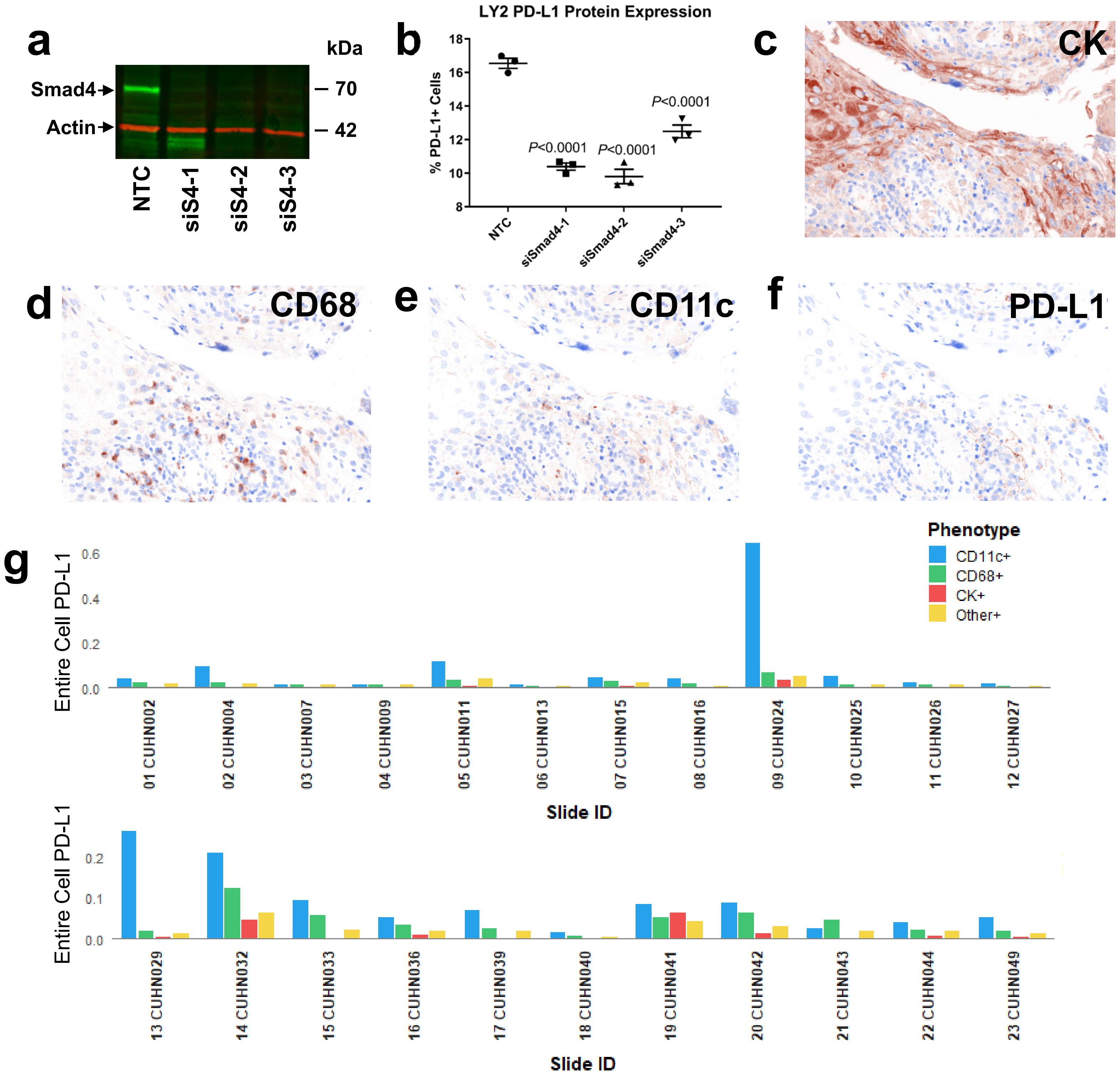

Supplementary Figure 3: LY2 PD-L1 expression requires Smad4, and human HNSCC PD-L1 expression is primarily on stromal cells.

a Smad4 protein (green, 70kDa), and actin protein (red, 42kDa) in LY2 cells treated with a non-template control (NTC), or three different siRNAs against Smad4 (siS4-1-3). **b** Percentage of PD-L1$^+$ cells in LY2 cell lines from **a** as quantified by flow cytometry *(n*=3 per group)*;* multiple comparisons against the NTC were performed by one-way ANOVA and post-hoc comparisons were performed by Dunnett's multiple comparisons test with *P* values as shown. **c** Cytokeratin expression in the representative tumor image from **Fig. 2f**. **d** CD68 expression in the representative tumor image from **Fig. 2f**. **e** CD11c expression in the representative tumor image from **Fig. 2f**. **f** PD-L1 expression in the representative tumor image from **Fig. 2f**. **g** Entire-cell PD-L1 expression scoring for all 23 individual human HNSCC tumor samples, divided into CK$^+$, CD68$^+$, CD11c$^+$ and Other$^+$ phenotypes. **c**-**f** were generated using the pathology view function of InForm software, and all error bars represent the SEM.

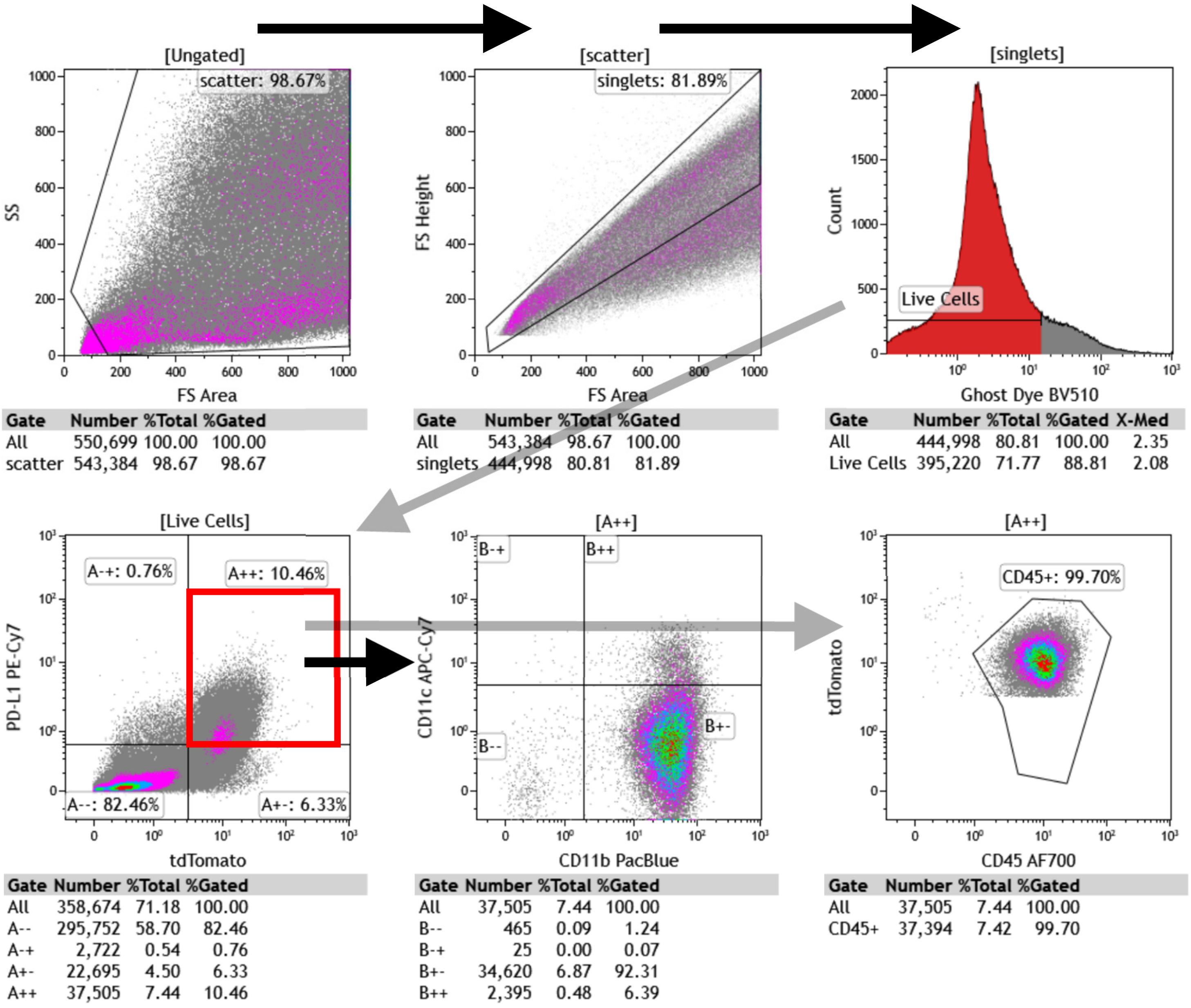

Supplementary Figure 4: Gating strategy for Fig. 2c, d.

Non-debris cells gated from side scatter and forward scatter area, singlets gated by forward scatter height and area, live cells gated by Ghost Dye Violet 510 exclusion, and then tdTomato expression was plotted against PD-L1 expression. CD11c expression was plotted against CD11b expression for $tdTomato^{+}PD\text{-}L1^{+}$ cells, which were then confirmed to be $CD45^{+}$.

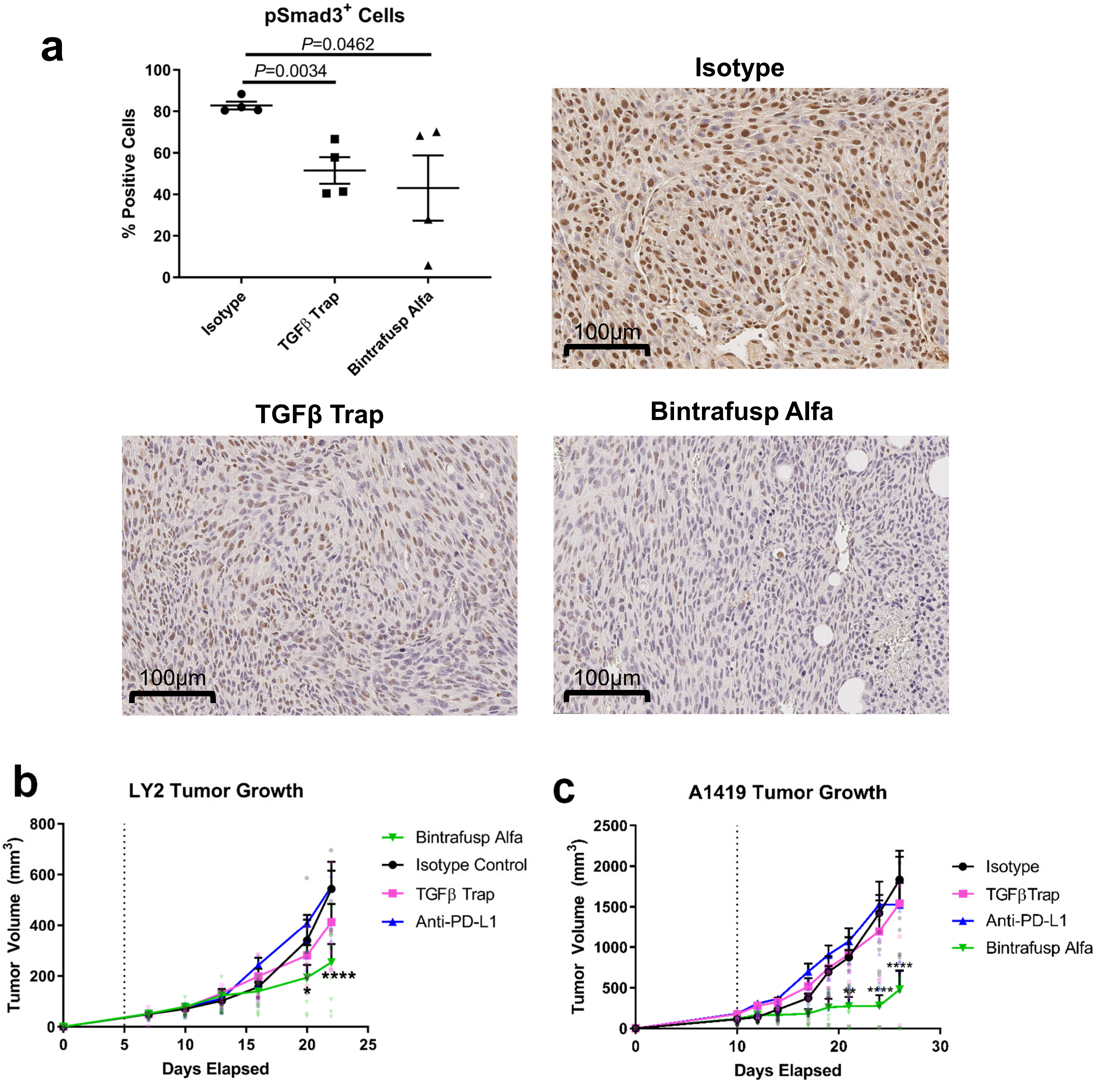

Supplementary Figure 5: LY2 and A1419 tumor growth after treatment with bintrafusp alfa.

a Quantification of pSmad3-positive cells in A223 tumors 48 hours after treatment with isotype control (circles), TGF trap (squares), or bintrafusp alfa (triangles) as in **Fig. 3**, and representative IHC staining images for each (*n*=4 per group). **b** Tumor growth of buccal LY2 tumors in balb/c mice treated with isotype control (black circles, *n*=6), TGFβ trap control (pink squares, *n*=7), anti-PD-L1 (blue triangles, *n*=7), or bintrafusp alfa (green triangles, *n*=7). **c** Tumor growth of subcutaneous A1419 tumors in C57BL/6 mice treated with isotype control (black circles, *n*=7), TGFβ trap control (pink squares, *n*=7), anti-PD-L1 (blue triangles, *n*=8), or bintrafusp alfa (green triangles, *n*=8). Differences between treatments were calculated by 2-way ANOVA using Tukey's correction for multiple comparisons; significant differences between bintrafusp alfa (green) and isotype control (black) are indicated with * for P=0.0218, ** for P=0.0071, and **** for P<0.0001, and all error bars represent the SEM.

Supplementary Table 1: List of antibody targets, their respective clone, and heavy metal conjugate used for CytoF analysis.

Target	Clone	Conjugate
CD45	30-F11	89Y
CD11b	M1/70	148Nd
CD11c	N418	142Nd
CD19	6D5	149Sm
CD4	RM4-5	172Yb
CD44	IM7	171Yb
CD62L	MEL-14	160Gd
CD25	3C7	151Eu
CD3ε	145-2C11	152Sm
TCRβ	H57-597	143Nd
CD8a	53-6.7	168Er
B220	RA3-6B2	176Yb
Arg1	Poly	166Er
Ly6C	HK1.4	150Nd
F4/80	BM8	146Nd
EpCAM	G8.8	165Ho
iNos	CXNFT	161Dy
Ly6G	1A8	141Pr
NK1.1	PK136	170Er
PD-1	RMP1-30	159Tb
PD-L1	10f.9G2	153Eu
CD69	H1.2F3	145Nd
MHC-II	M5/114.15.2	174Yb

Supplementary Table 2: List of infiltrating immune cell phenotypes and the markers used to define them.

**Cell Type**	**Definition (All $CD45^{+}$)**
B Cells	$CD19^{+}$ $B220^{+}$
Dendritic Cells	$CD11c^{+}$ $F4/80^{-}$ $Ly6C^{-}$ $Ly6G^{-}$ $MHC\text{-}II^{+}$
Inflammatory Monocytes	$CD11b^{+}$ $Ly6C^{high}$ $Ly6G^{-}$ $MHCII^{-}$ $F4/80^{+}$ $CD11c^{-}$
Resident Monocytes	$CD11b^{+}$ $Ly6C^{low}$ $Ly6G^{-}$ $MHCII^{-}$ $F4/80^{+}$ $CD11c^{-}$
Monocytic MDSCs	$CD11b^{+}$ $Ly6C^{high}$ $Ly6G^{-}$
Polymorphonuclear MDSCs	$CD11b^{+}$ $Ly6C^{low}$ $Ly6G^{+}$
M1 Macrophages	$CD11b^{+}$ $F4/80^{+}$ $Ly6G/C^{-}$ $iNos^{+}$ $MHCII^{high}$
M2 Macrophages	$CD11b^{+}$ $F4/80^{+}$ $Ly6G/C^{-}$ $Arg1^{+}$ $MHCII^{low}$
Tregs	$CD3\varepsilon^{+}$ $TCR\beta^{+}$ $CD4^{+}$ $CD25^{+}$
NK Cells	$CD3\varepsilon^{-}$ $TCR\beta^{-}$ $NK1.1^{+}$
CD4 T Cells	$CD3\varepsilon^{+}$ $TCR\beta^{+}$ $CD4^{+}$
CD8 T Cells	$CD3\varepsilon^{+}$ $TCR\beta^{+}$ $CD8a^{+}$

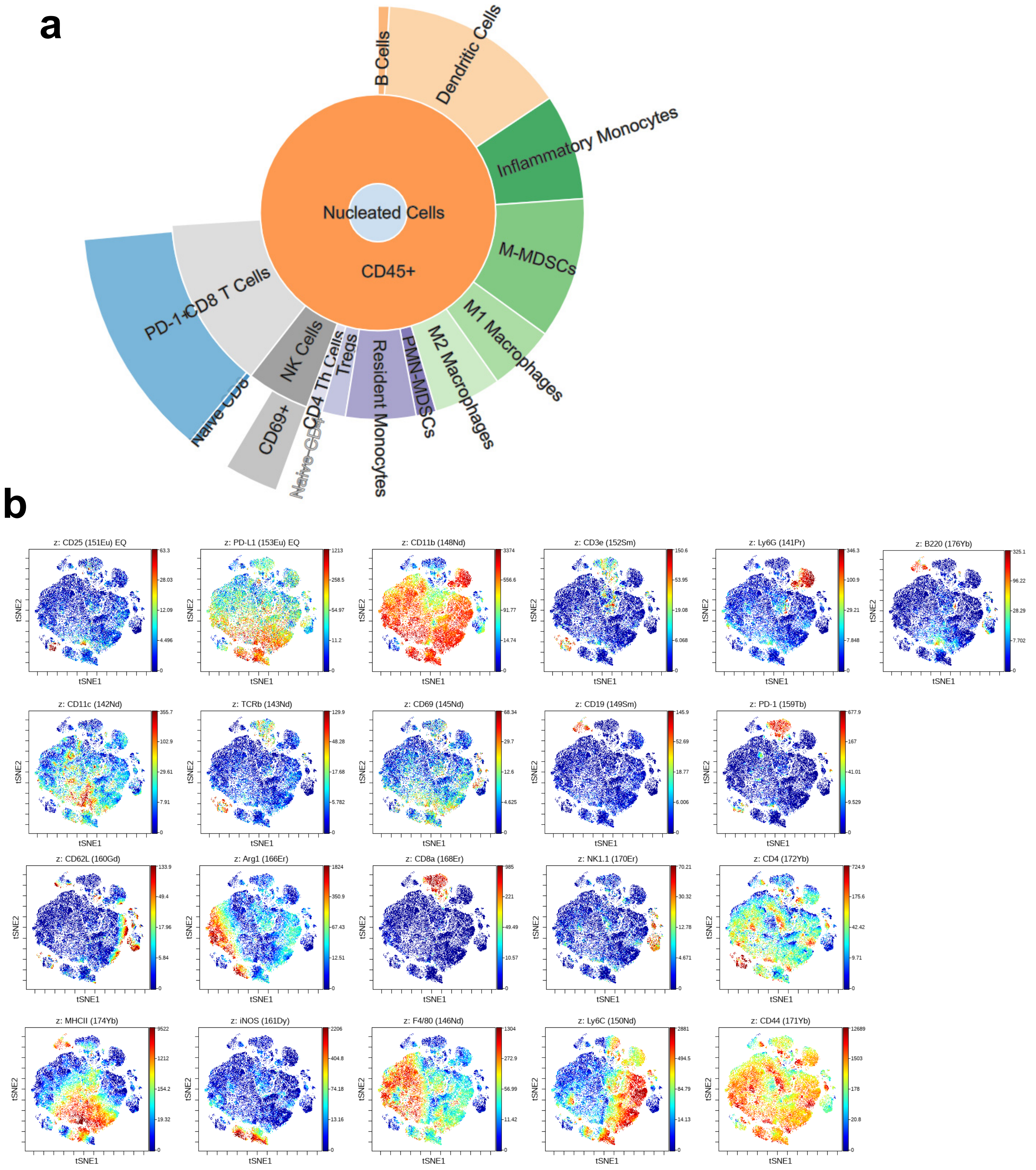

Supplementary Figure 6: Example population profile after mass cytometry and immune cell profiling.

a Representative population tree of live, nucleated, CD45$^+$ cells in one isotype-treated tumor. **b** tSNE plots for each individual marker used in concert with **Supplementary Table 2** to generate the population tree from **a**.

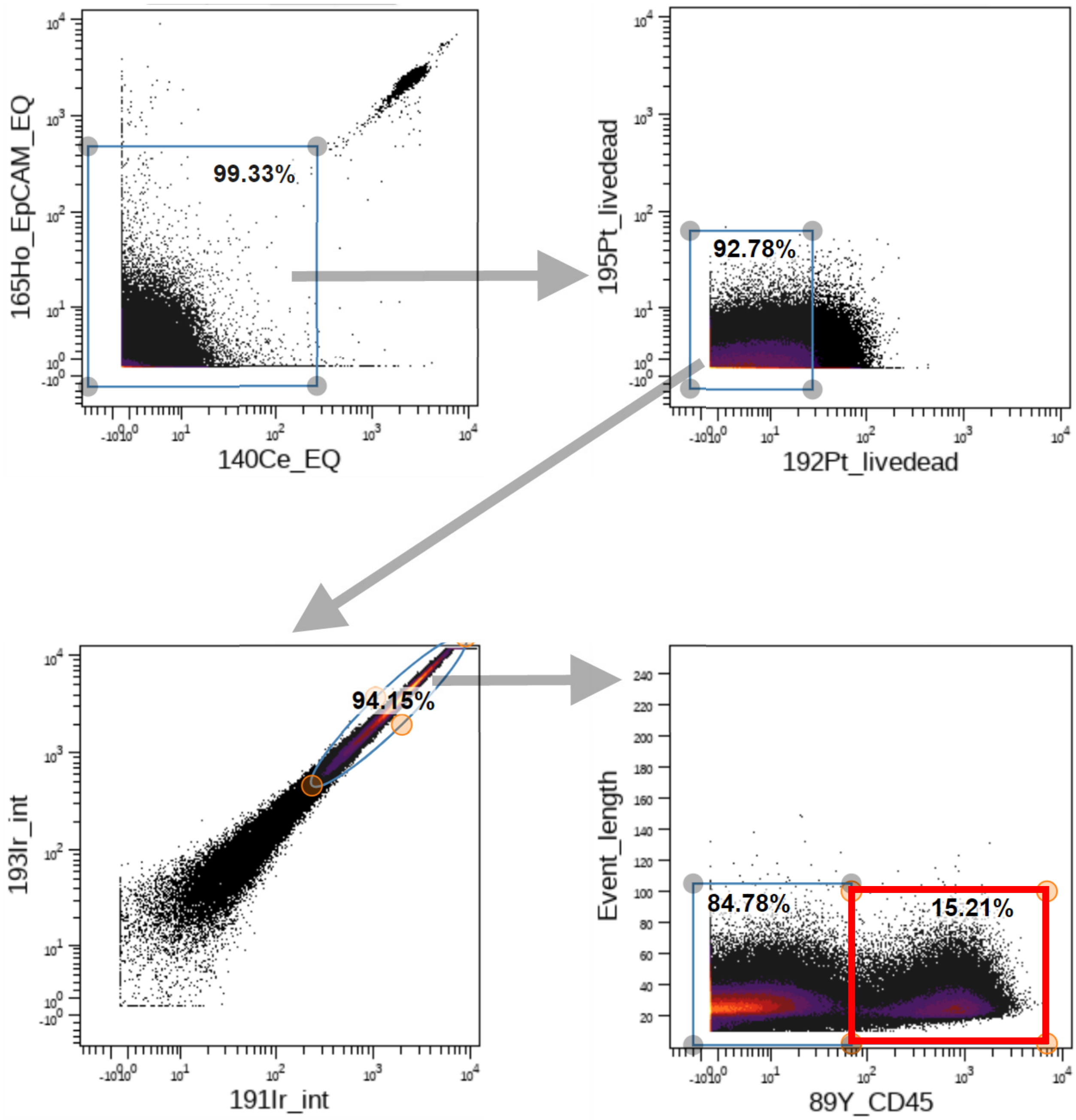

Supplementary Figure 7: Gating strategy for Figs. 4a, 4e, and Supplementary Figs. 5a, 5b, 8a-c.

Equalizer beads were first exluded, then cells excluding Cisplatin live/dead stain were gated, followed by cells staining positive for iridium intercalator DNA stain, from which $CD45^{+}$ cells were selected for viSNE analysis.

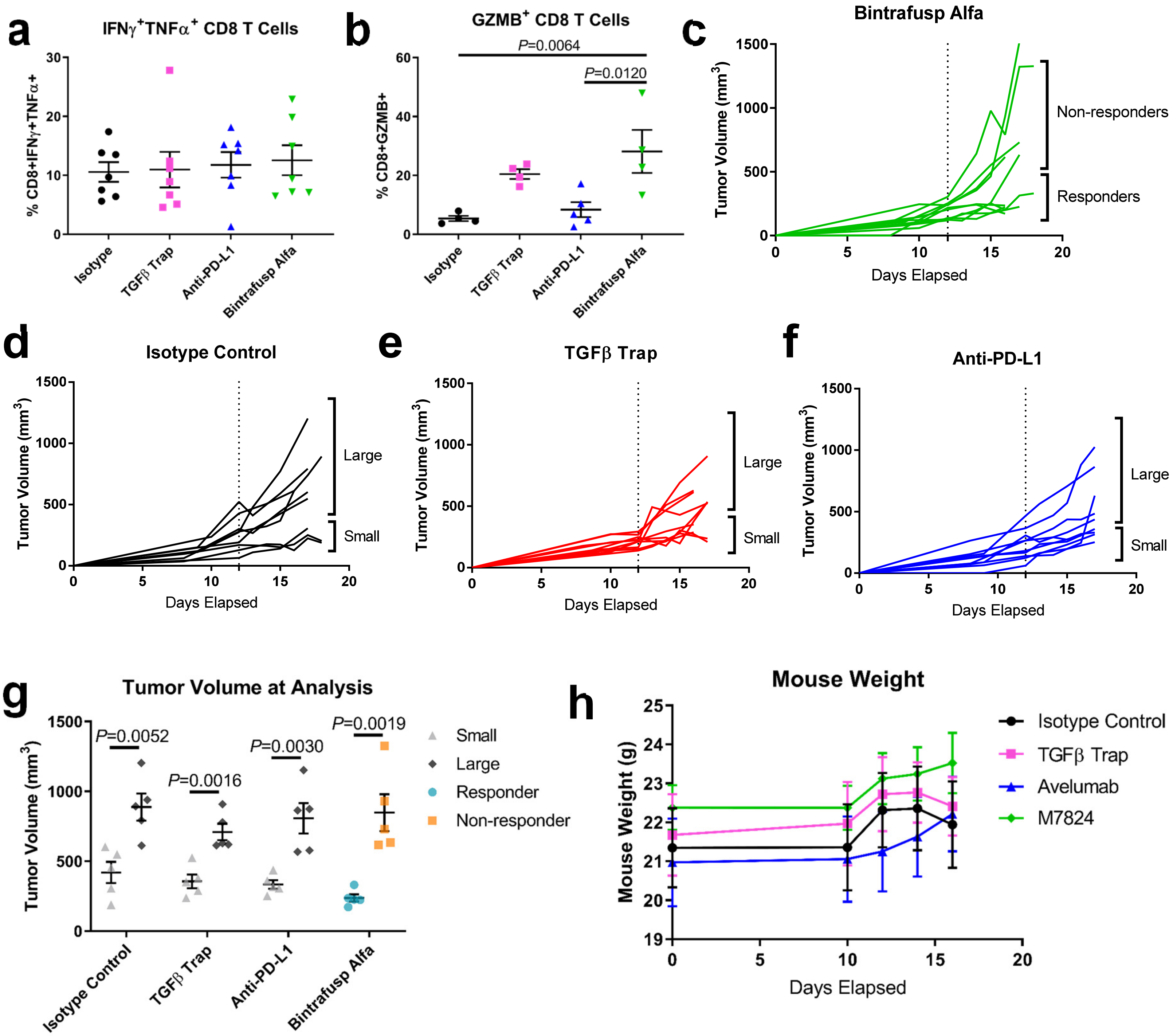

Supplementary Figure 8: Individual A223 tumor tracks and size group differentiation for flow cytometric analysis.

Tumor digests from **Fig. 4a** were treated for 4 hours with brefeldin-A, ionomycin, and PMA, and their **a** $CD8^{+}IFN\alpha^{+}TNF\alpha^{+}$ and **b** $CD8^{+}$Granzyme B^{+} cells were quantified by flow cytometry (*n*=7 per group). Individual tumor tracks of mice bearing A223 tumors treated with **c** bintrafusp alfa, **d** isotype control, **e** TGFβ trap, and **f** anti-PD-L1 that were harvested for mass cytometric analysis in **Fig. 4** (*n*=10 per group). Tumors were sub-grouped into "large" and "small" size or "responder" and "non-responder" populations as indicated. **g** Differences in average tumor volume for groups identified in **c-f** with significant differences as indicated *(n*=5 per treatment). **h** Mouse weights on mice analyzed in **Fig. 4** and **Supplementary Fig. 8** over the course of treatment (*n*=10 per group). Unpaired two-tailed t tests were performed for **g**. A one-way ANOVA with multiple comparisons corrected for by Tukey's method was performed for **g** and a two-way ANOVA was performed for **h** with significant differences as indicated. All error bars represent the SEM**.**

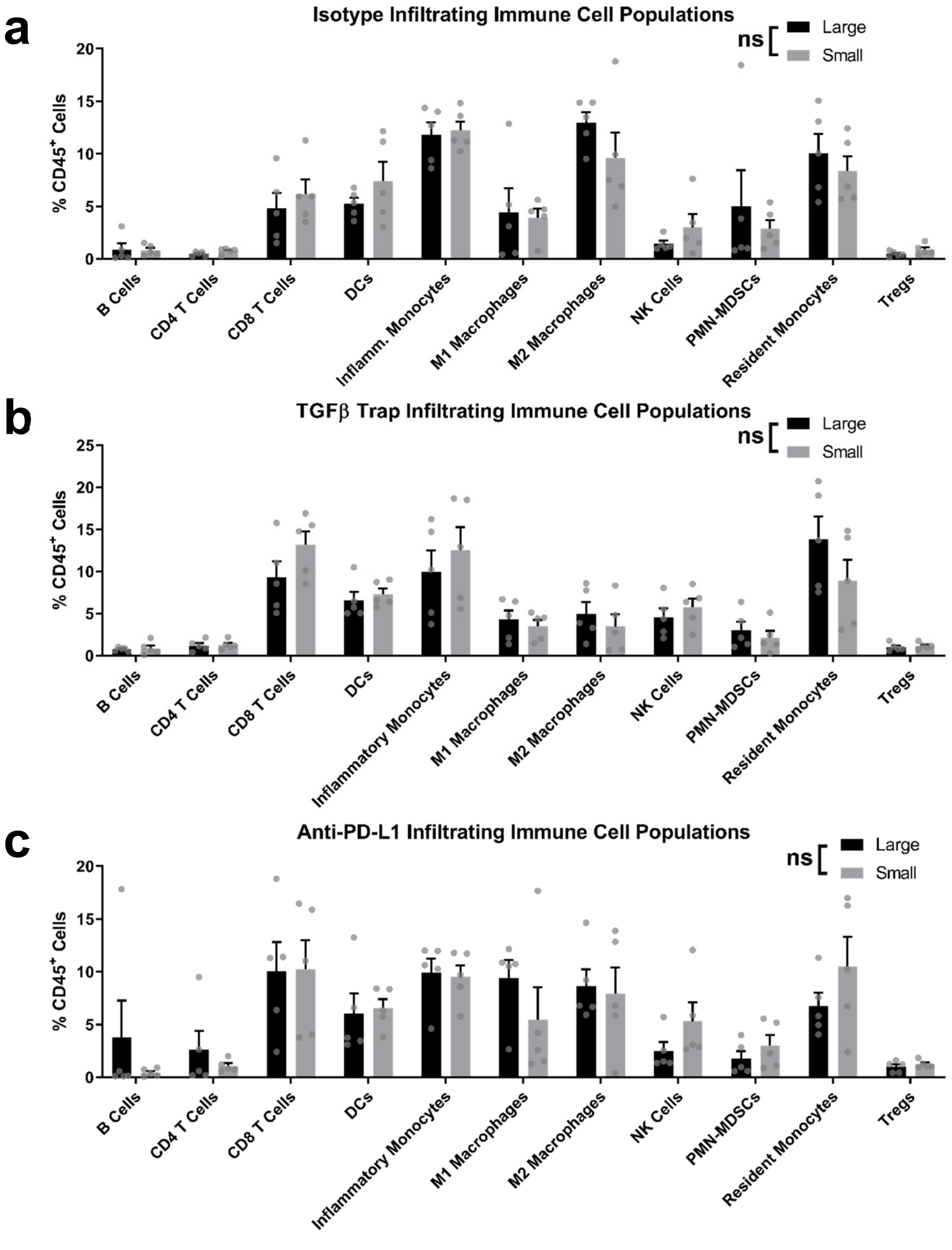

Supplementary Figure 9: Infiltrating immune cell populations do not change based on tumor size or treatment without differentiating between responders and non-responders.

a Infiltrating immune cell populations of large (black, *n*=5) and small (gray, *n*=5) tumor subpopulations of isotype-treated mice from **Fig. 4a** after mass cytometric analysis and clustering. **b** Infiltrating immune cell populations of large (black, *n*=5) and small (gray, *n*=5) tumor subpopulations of TGFβ trap-treated mice from **Fig. 4a** after mass cytometric analysis and clustering. **c** Infiltrating immune cell populations of large (black, *n*=5) and small (gray, *n*=5) tumor subpopulations of anti-PD-L1-treated mice from **Fig. 4a** after mass cytometric analysis and clustering. Multiple comparisons were performed by 2-way ANOVA with no significant differences between groups for **a** (*P*=0.6885, F=0.7363, df = 10), **b** (*P*=0.2670, F=1.258, df=10), and **c** (*P*=0.6370, F=0.7914, df=10). Post-hoc analyses were performed using Sidak's multiple comparisons test with no significantly altered categories found, and all error bars represent the SEM.

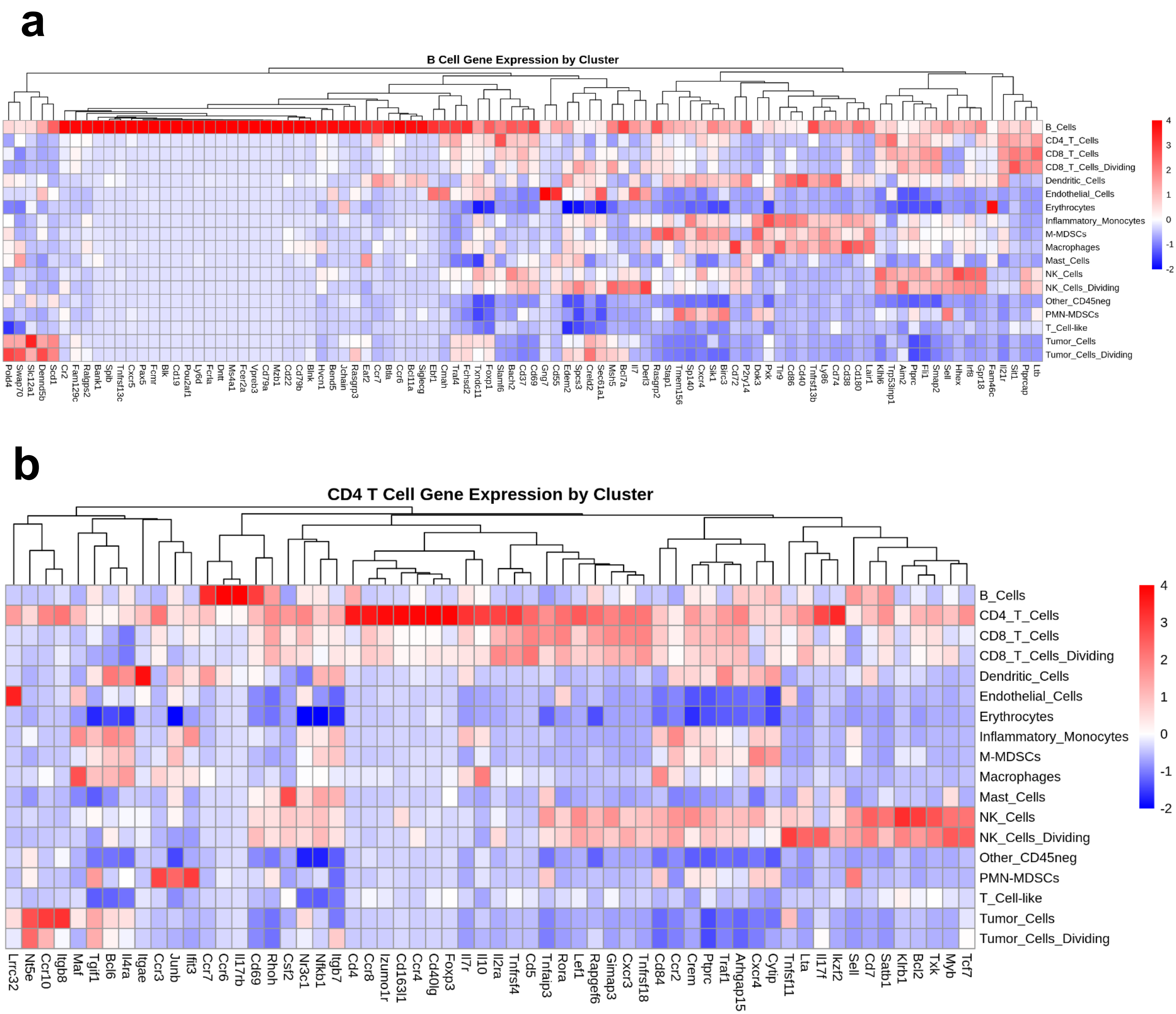

Supplementary Figure 10: UMAP cluster expression of known B and CD4 T cell markers.

a Gene expression of the Panglao database “B Cells” and “B Cells Memory” categories across all UMAP clusters. **b** Gene expression of the Panglao database “T Helper Cells” category across all UMAP clusters.

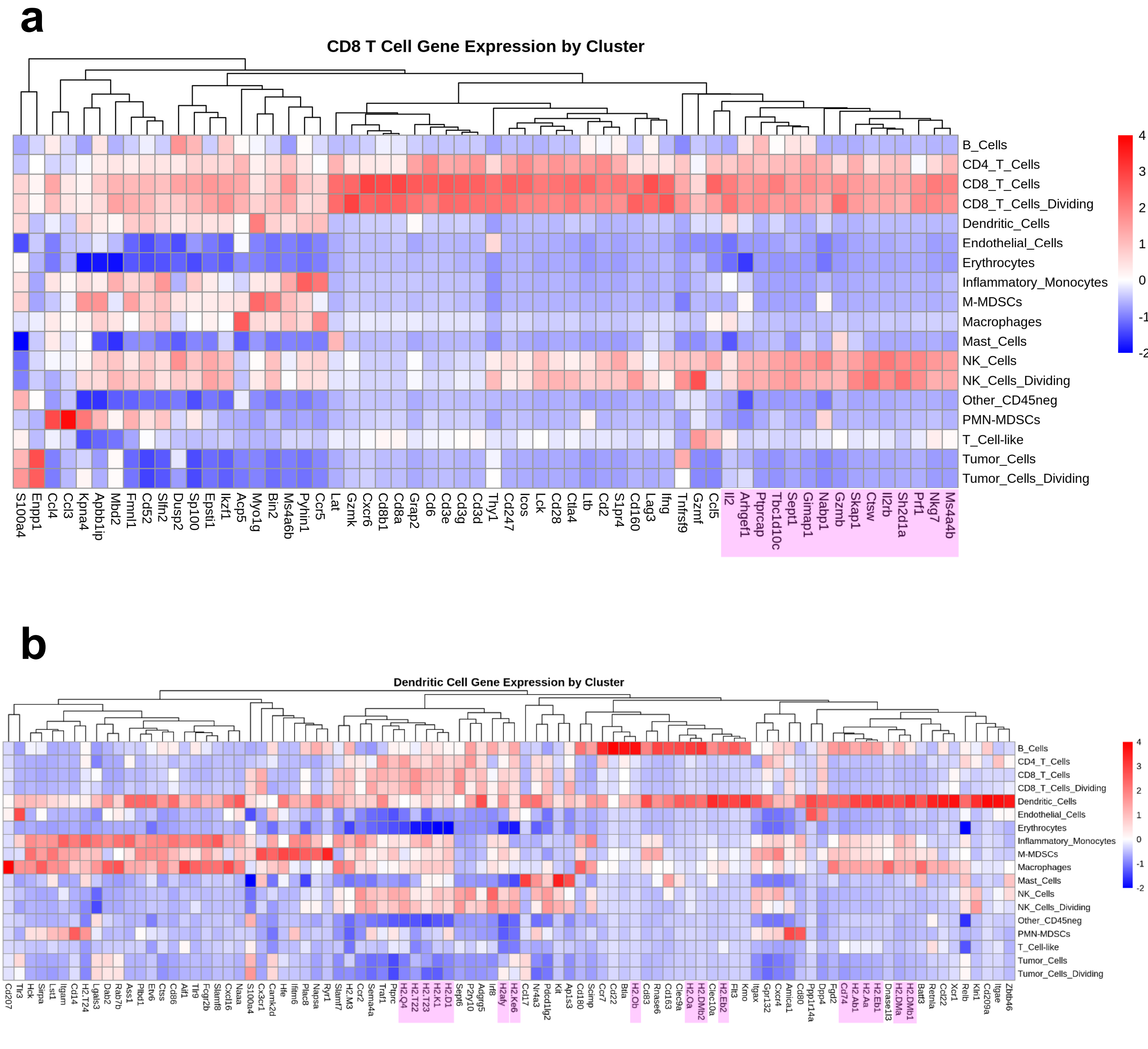

Supplementary Figure 11: UMAP cluster expression of known CD8 and dendritic cell markers.

a Gene expression of the Panglao database “T Memory” and “T Cytotoxic Cells” categories across all UMAP clusters, with additional curated genes as marked. **b** Gene expression of the Panglao database “Dendritic Cells” category across all UMAP clusters, with additional curated genes highlighted.

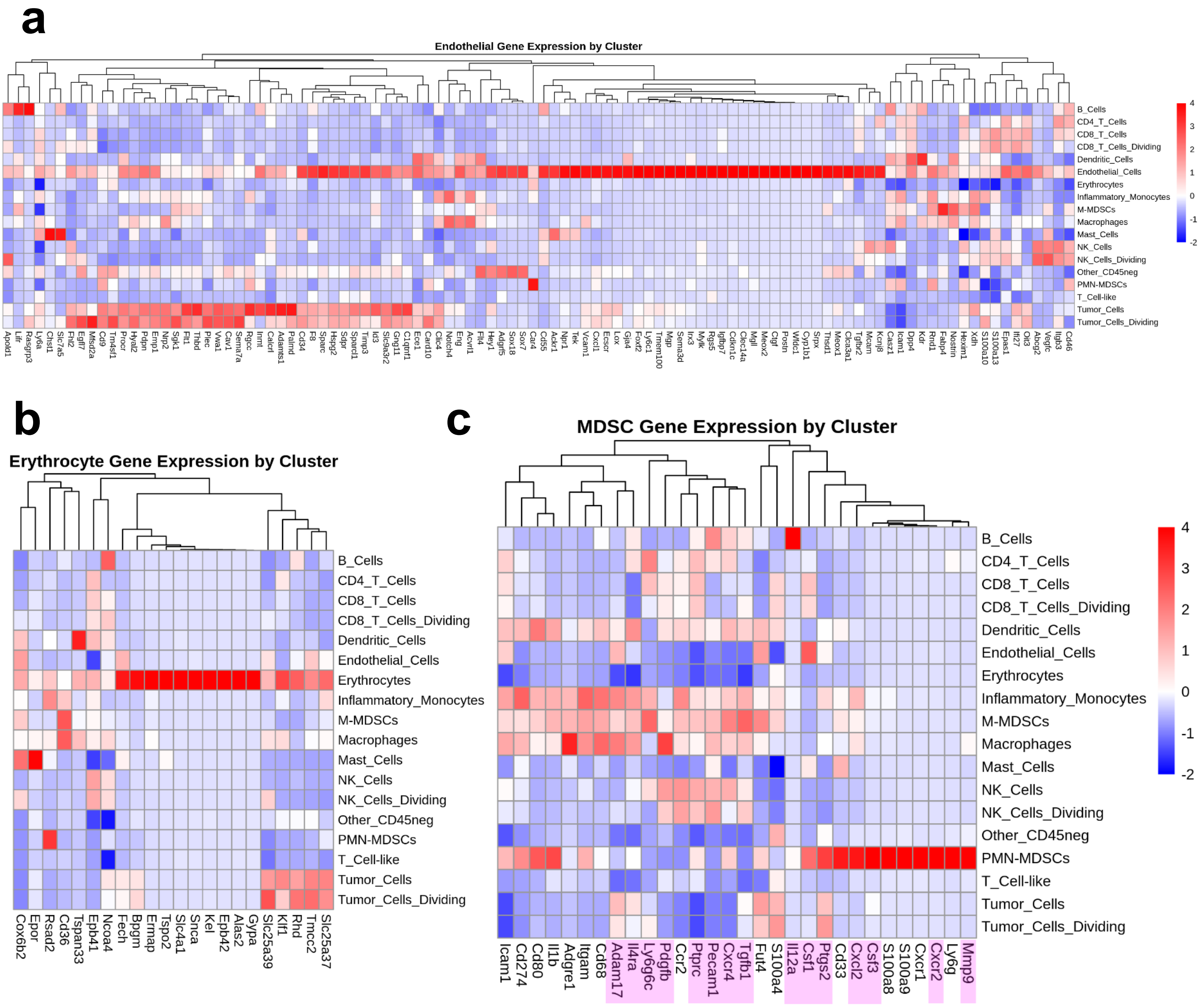

Supplementary Figure 12: UMAP cluster expression of known endothelial cell, erythrocyte, and MDSC markers.

a Gene expression of the Panglao database “Endothelial Cells” category across all UMAP clusters. **b** Gene expression of the Panglao database “Erythroid-like and erythroid percursor cells” category across all UMAP clusters. **c** Gene expression of the Panglao database “Myeloid-derived suppressor cells” category across all UMAP clusters, with additional curated genes highlighted.

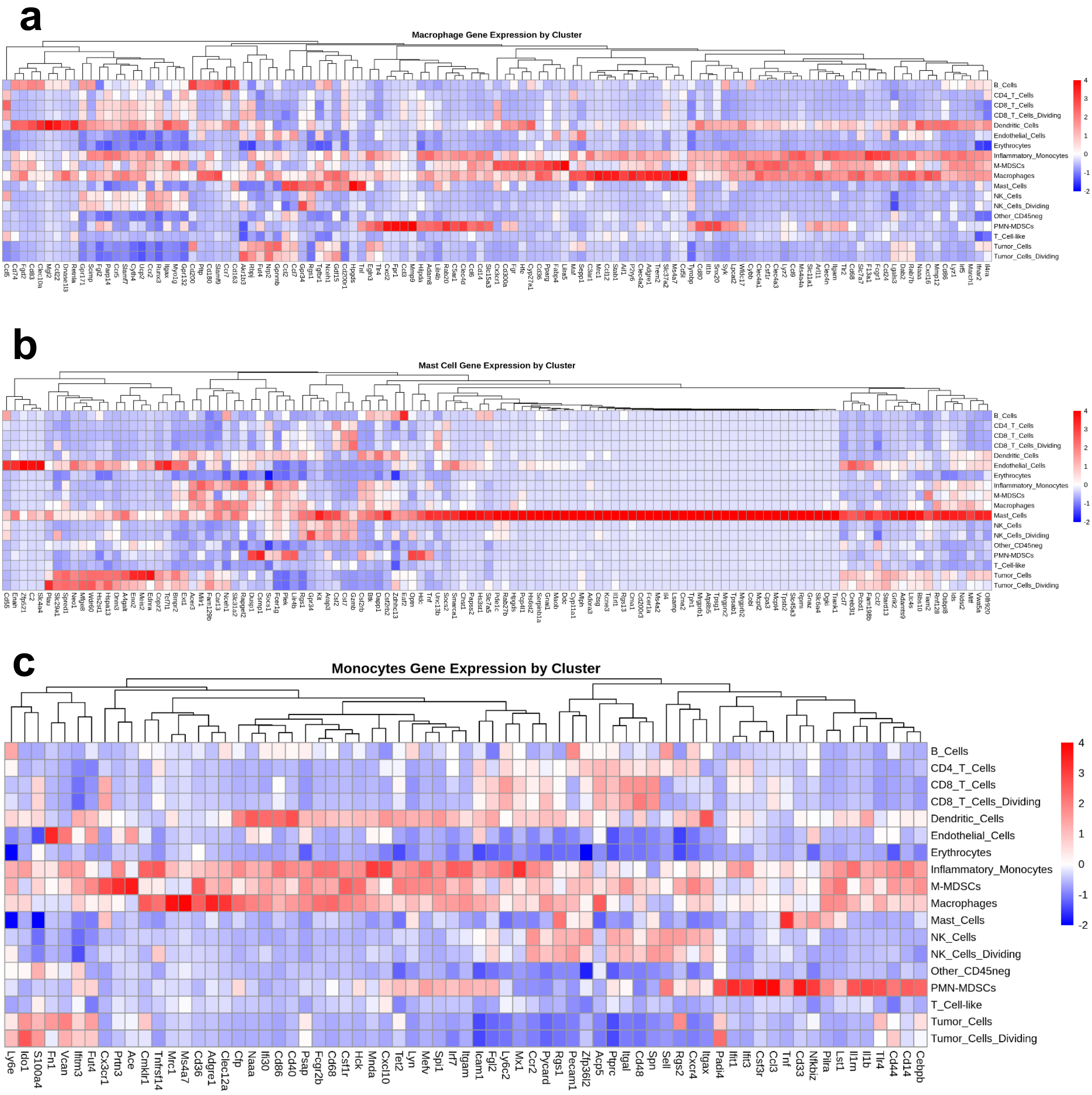

Supplementary Figure 13: UMAP cluster expression of known macrophage, mast cell, and monocyte markers.

a Gene expression of the Panglao database "Macrophages" category across all UMAP clusters. **b** Gene expression of the Panglao database "Mast Cells" category across all UMAP clusters. **c** Gene expression of the Panglao database "Monocytes" category across all UMAP clusters.

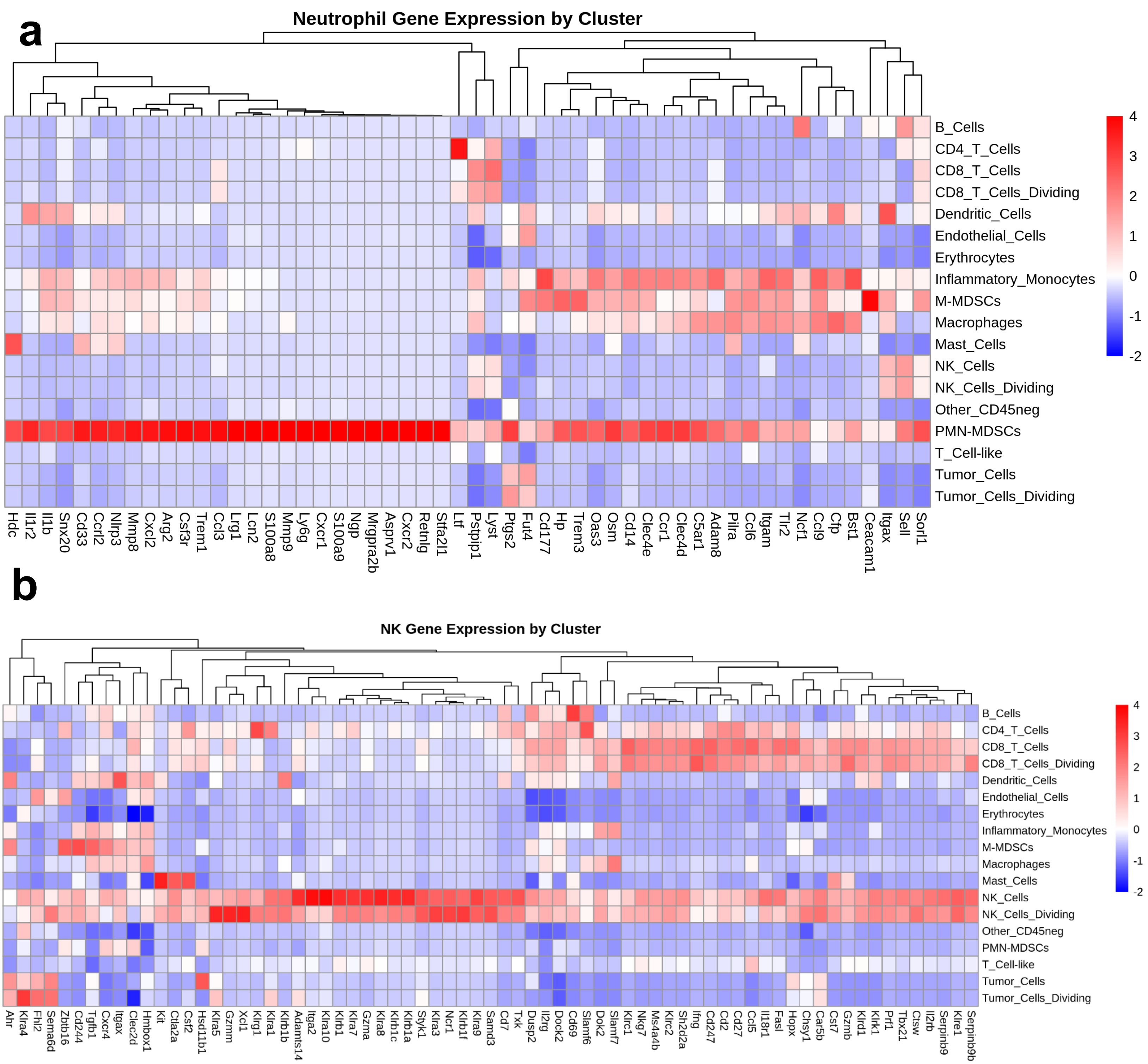

Supplementary Figure 14: UMAP cluster expression of neutrophil and NK cell markers.

a Gene expression of the Panglao database “Neutrophils” category across all UMAP clusters. **b** Gene expression of the Panglao database “NK Cells” category across all UMAP clusters.

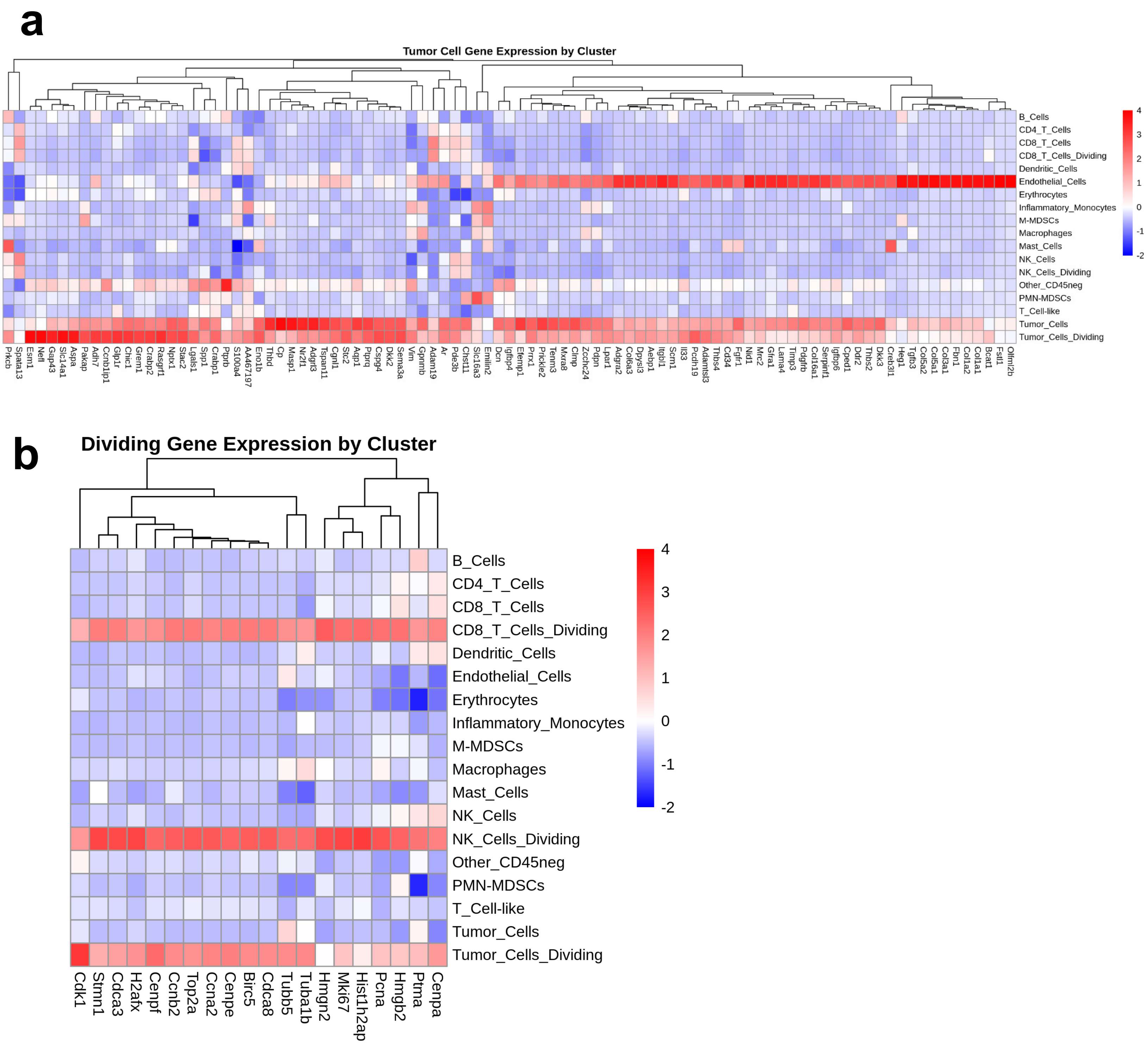

Supplementary Figure 15: UMAP cluster expression of A223 tumor cell markers and dividing cell markers across all clusters

a Gene expression of A223 tumor cell markers that were highly expressed in bulk RNA sequencing of A223 cells *in vitro*. **b** Gene expression of dividing cell markers across all clusters.

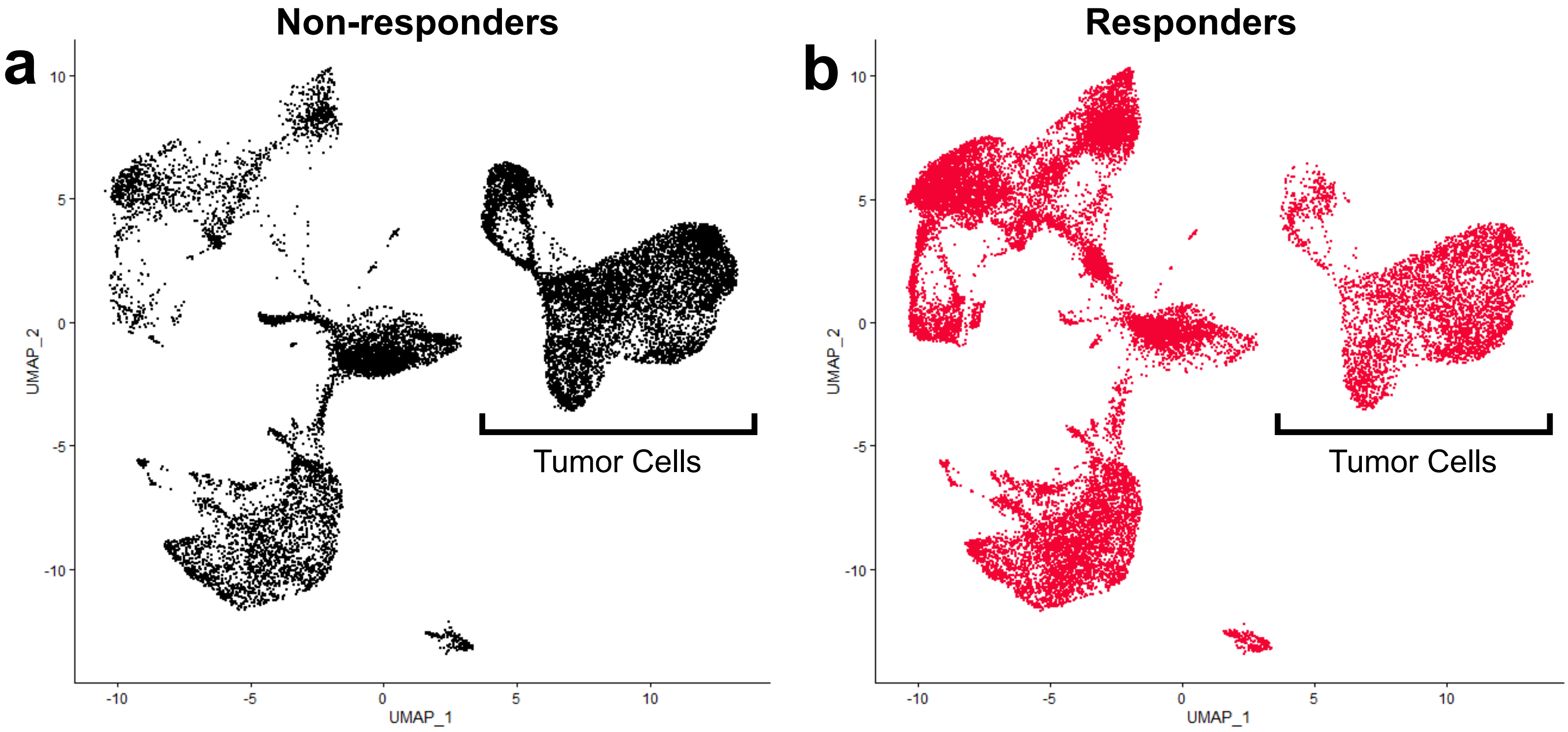

Supplementary Figure 16: UMAP clusters split between non-responder and responder statuses.

UMAP clusters from **Fig. 5a** split into **a** non-responders and **b** responders with clusters representing tumor cells as indicated.

a

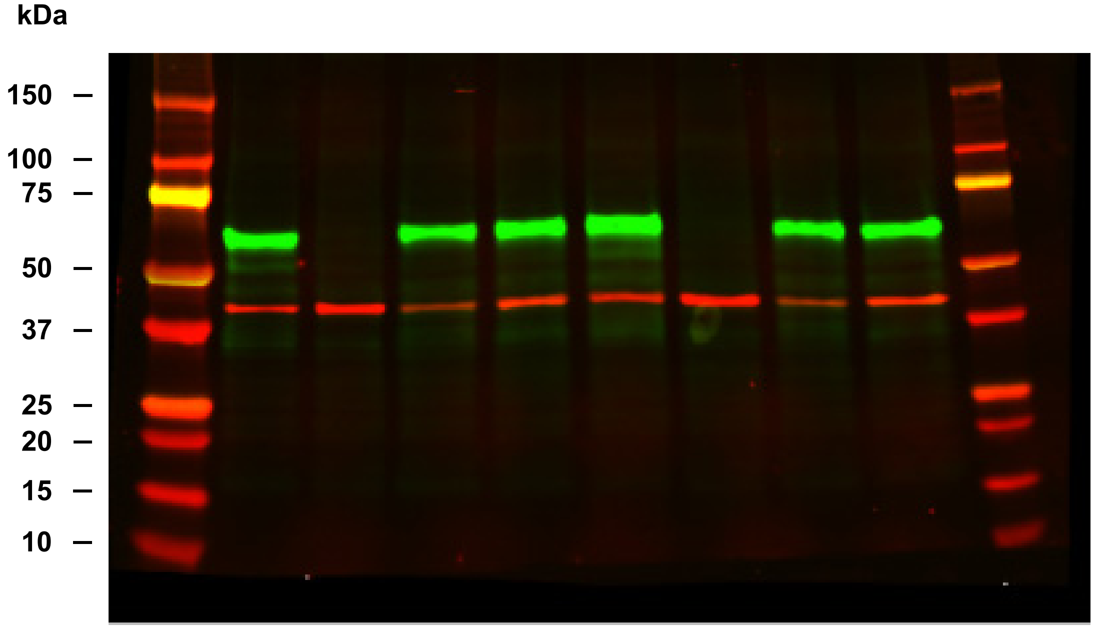

b

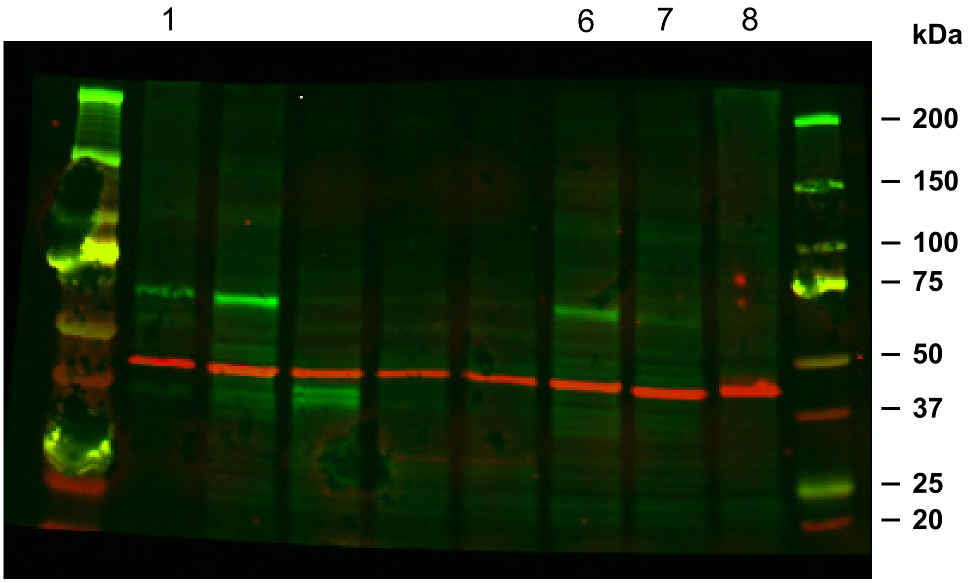

Supplementary Figure 17: Raw western blot images from Supplementary Figs. 1 and 3

a Raw western blot image used in **Supplementary Fig. 1**, with Smad4 stained in green and actin stained in red. Two independent biological replicates were loaded (4 lanes each), with the left one used for the figure. **b** Raw western blot image used for **Supplementary Fig. 3**, with Smad4 stained in green and actin stained in red. Two independent biological replicates were loaded (4 lanes each), with the left one used for the figure. Lanes 1 is A549 cell lysate that was not referenced in this publication, lanes 6 and 7 are off-target siRNAs against Smad4, and lane 8 is A223 cell lysate as a negative control.
