# Supplementary material for: Distinct immune microenvironment profiles of therapeutic responders emerge in combined TGFβ/PD-L1 blockade-treated squamous cell carcinoma
Source: Commun Biol. 2021 Aug 25;4:1005. doi: 10.1038/s42003-021-02522-2 (PMC8387430; doi:10.1038/s42003-021-02522-2)
Supplement: Supplementary file 5 — Reporting Summary [file 42003_2021_2522_MOESM5_ESM.pdf]

nature portfolio

Corresponding author(s): Xiao-Jing Wang, Jing H. Wang

Last updated by author(s): Jul 22, 2021

Reporting Summary

Nature Portfolio wishes to improve the reproducibility of the work that we publish. This form provides structure for consistency and transparency in reporting. For further information on Nature Portfolio policies, see our Editorial Policies and the Editorial Policy Checklist.

Statistics

For all statistical analyses, confirm that the following items are present in the figure legend, table legend, main text, or Methods section.

n/a	Confirmed	
☐	☒	The exact sample size (*n*) for each experimental group/condition, given as a discrete number and unit of measurement
☐	☒	A statement on whether measurements were taken from distinct samples or whether the same sample was measured repeatedly
☐	☒	The statistical test(s) used AND whether they are one- or two-sided<br>*Only common tests should be described solely by name; describe more complex techniques in the Methods section.*
☒	☐	A description of all covariates tested
☐	☒	A description of any assumptions or corrections, such as tests of normality and adjustment for multiple comparisons
☐	☒	A full description of the statistical parameters including central tendency (e.g. means) or other basic estimates (e.g. regression coefficient) AND variation (e.g. standard deviation) or associated estimates of uncertainty (e.g. confidence intervals)
☐	☒	For null hypothesis testing, the test statistic (e.g. *F*, *t*, *r*) with confidence intervals, effect sizes, degrees of freedom and *P* value noted<br>*Give P values as exact values whenever suitable.*
☒	☐	For Bayesian analysis, information on the choice of priors and Markov chain Monte Carlo settings
☒	☐	For hierarchical and complex designs, identification of the appropriate level for tests and full reporting of outcomes
☒	☐	Estimates of effect sizes (e.g. Cohen's *d*, Pearson's *r*), indicating how they were calculated

Our web collection on statistics for biologists contains articles on many of the points above.

Software and code

Policy information about availability of computer code

Data collection	MSI images were acquired with Phenochart v1.0.12 (Akoya Biosciences).
Data analysis	MSI images were analyzed with inForm v2.4.10 and PhenoptrReports v0.2.8 (Akoya Biosciences). Flow cytometry was analyzed with Kaluza Analysis v2.1 (Beckman Coulter). CyTOF mass cytometry was analyzed using CytoBank v7 (Beckman Coulter). Single-cell RNA sequencing data was analyzed using Seurat v3.1.5 and visualized using pheatmap v1.0.12. Statistical analyses and visualizations were performed with GraphPad v7.02 (Graphpad Software, Inc.).

For manuscripts utilizing custom algorithms or software that are central to the research but not yet described in published literature, software must be made available to editors and reviewers. We strongly encourage code deposition in a community repository (e.g. GitHub). See the Nature Portfolio guidelines for submitting code & software for further information.

Data

Policy information about availability of data

All manuscripts must include a data availability statement. This statement should provide the following information, where applicable:

- Accession codes, unique identifiers, or web links for publicly available datasets
- A description of any restrictions on data availability
- For clinical datasets or third party data, please ensure that the statement adheres to our policy

The data that support the findings of this study are available from the corresponding authors upon request. Single-cell RNA sequencing data and metadata is available via NCBI GEO (accession GSE161370). Raw western blot images are available in Supplementary Fig. 17 and the source data for all figures is available in Supplementary Data 1.

Field-specific reporting

Please select the one below that is the best fit for your research. If you are not sure, read the appropriate sections before making your selection.

☒ Life sciences ☐ Behavioural & social sciences ☐ Ecological, evolutionary & environmental sciences

For a reference copy of the document with all sections, see nature.com/documents/nr-reporting-summary-flat.pdf

Life sciences study design

All studies must disclose on these points even when the disclosure is negative.

Sample size	Sample sizes were chosen based on previously published experiments and experience with the size distribution of the tumor cell lines presented here.
Data exclusions	No data were excluded from analyses.
Replication	A223 tumor growth assays are representative of three biologically independent experiments. LY2 and A1419 growth assays are representative of two biologically independent experiments. Mass cytometry assays are pooled results from two biologically independent experiments that were representative of four independent experiments. Single-cell RNA sequencing is presented as the combination of two biologically independent experiments.
Randomization	Tumors were sorted by size and then randomly assigned into treatment groups by groups of 4 to maintain a generally similar mean tumor volume in each cohort.
Blinding	Tumor growth volume was measured without knowledge of treatment groups. Tumor growth data was entered into a blind spreadsheet and then un-blinded after the experiment conclusion.

Reporting for specific materials, systems and methods

We require information from authors about some types of materials, experimental systems and methods used in many studies. Here, indicate whether each material, system or method listed is relevant to your study. If you are not sure if a list item applies to your research, read the appropriate section before selecting a response.

Materials & experimental systems

n/a	Involved in the study
☐	☒ Antibodies
☐	☒ Eukaryotic cell lines
☒	☐ Palaeontology and archaeology
☐	☒ Animals and other organisms
☒	☐ Human research participants
☒	☐ Clinical data
☒	☐ Dual use research of concern

Methods

n/a	Involved in the study
☒	☐ ChIP-seq
☐	☒ Flow cytometry
☒	☐ MRI-based neuroimaging

Antibodies

Antibodies used

Anti-Smad4 (mouse/rat/human) - AbCam cat. Ab40759, clone EP618Y,
Anti-beta-Actin - Santa Cruz BioTech cat. SC-47778, clone C4
Anti-PD-L1 (human) - Cell Signaling Technologies cat. 13684S, clone E1L3N, lot 13.
Anti-CD11c (human) - Sigma Cell Marque cat. 111M-15, clone 5D11, lot CMC11121020
Anti-CD68 (human) - Dako cat. M0814, clone KP1
Anti-pan-cytokeratin (human) - Dako cat. M351501-2, clone AE1/AE3
Anti-CD16/32 (mouse) - BD Biosciences cat. 553142, clone 2.4G2, lot 7248907
Anti-CD45 (mouse) - BioLegend cat. 103127, clone 30-F11, lot B240371
Anti-PD-L1 (mouse) - BioLegend cat. 124313, clone 10F.9G2, lot B223286
Anti-CD11b (mouse) - BioLegend cat. 101223, clone M1/70, lot B247650
Anti-CD11c (mouse) - BioLegend cat. 117323, clone N418, lot B263880
Anti-CD8 (mouse) - BioLegend cat. 100747, clone 53-6.7
Anti-CD45 (mouse) - BD Bioscience cat. 564279, clone 30-F11
Anti-CD4 (mouse) - BioLegend cat. 100563, clone RM4-5
Anti-IFN-gamma (mouse) - eBioscience cat. 12-7311-41, clone XMG1.2
Anti-TNF-alpha (mouse) - BioLegend cat. 506307, clone MP6-XT22
Anti-CD45 (mouse) - Fluidigm cat. 3089005B, clone 30-F11
Anti-CD11b (mouse) - Fluidigm cat. 3148003B, clone M1/70
Anti-CD11c (mouse) - Fluidigm cat. 3142003B, clone N418

	Anti-CD19 (mouse) - Fluidigm cat. 3149002B, clone 6D5<br>Anti-CD4 (mouse) - Fluidigm cat. 3172003B, clone RM4-5<br>Anti-CD44 (human/mouse) - Fluidigm cat. 3171003B, clone IM7<br>Anti-CD62L (mouse) - Fluidigm cat. 3160008B, clone MEL-14<br>Anti-CD25 (mouse) - Fluidigm cat. 3151007B, clone 3C7<br>Anti-CD3ε (mouse) - Fluidigm cat. 3152004B, clone 145-2C11<br>Anti-TCR-beta (mouse) - Fluidigm cat. 3143010B, clone H57-597<br>Anti-CD8a (mouse) - Fluidigm cat. 3168003B, clone 53-6.7<br>Anti-B220 (mouse) - Fluidigm cat. 3176002B, clone RA3-6B2<br>Anti-Ly6G (mouse) - Fluidigm cat. 3141005B, clone 1A8<br>Anti-NK1.1 (mouse) - Fluidigm cat. 3170002B, clone PK136<br>Anti-Arg1 (human/mouse/rat) - Fluidigm cat. 3166023B, polyclonal<br>Anti-F4/80 (mouse) - Fluidigm cat. 3146008B, clone BM8<br>Anti-MHC-II (mouse) - Fluidigm cat. 3174003B, clone M5/114.15.2<br>Anti-EpCAM (mouse) - Fluidigm cat. 3165014B, clone G8.8<br>Anti-iNOS (mouse) - Fluidigm cat. 3161011B, clone CXNFT<br>Anti-PD-1 (mouse) - Fluidigm cat. 3159023B, clone RMP1-30<br>Anti-PD-L1 (mouse) - Fluidigm cat. 3153016B, clone 10F.9G2<br>Anti-CD69 (mouse) - Fluidigm cat. 3145005B, clone H1.2F3<br>Anti-Ly6C (mouse) - Biolegend cat. 128039, clone HK1.4<br>Anti-Granzyme B (mouse) - Biolegend cat. 372203, clone QA16A02<br>Anti-phospho-Smad3 (mouse) - Abcam cat. ab52903, clone EP823Y
Validation	Anti-Smad4 (mouse/rat/human) - manufacturer provided SH-SY5Y, Ramos, NIH/3T3, PC-12, HepG2, Jurkat, and Hap1 +/- Smad4 cell lines, and mouse embryo, skin, and lung tissue western blots, 94 citations.<br>Anti-beta-Actin - manufacturer provided HeLa, NIH/3T3, KNRK, Sol8, C32, ES-D3, F9, Jurkat, HL-60, DU145, MCF7, MDA-MB-231, HCT-116, HUV-EC-C, SUP-T1, NTERA-2 cl.D1, ES-D3, CCRF-CEM, U-698-M, Ramos cell lines and human andrenal gland western blots, 9189 citations.<br>Anti-PD-L1 (human) - manufacturer provided analysis of paraffin-embedded human sample tissue controls (NSCLC, human placenta, lung carcinoma, Karpas-299 cells, breast carcinoma, ovarian carcinoma), 233 citations.<br>Anti-CD11c (human) - manufacturer provided analysis of paraffin-embedded human sample tissue controls (hairy cell leukemia, bone marrow, and granulocytes), 5 citations.<br>Anti-CD68 (human) - 387 citations.<br>Anti-pan-cytokeratin (human) - 421 citations.<br>Anti-CD16/32 (mouse) - manufacturer provided evidence of blocking mouse splenocyte staining for CD90.2, and 19 citations.<br>All Fluidigm antibodies except Arg1, F4/80, MHC-II, EpCAM, iNOS, PD-1, PD-L1, CD69 - manufacturer provided mouse splenocyte staining data, 2 references.<br>Anti-Arg1 (human/mouse) - manufacturer provided HepG2 cell line and PBMC staining data, 2 references.<br>Anti-F4/80, anti-MHC-II (mouse) - manufacturer provided bone marrow staining, 2 references.<br>Anti-EpCAM (mouse) - manufacturer provided mouse ovary cell staining, 2 references.<br>Anti-iNOS (mouse) - manufacturer provided LPS-stimulated Raw 264.7 cell staining, 2 references.<br>Anti-PD-1, anti-PD-L1 (mouse) - manufacturer provided Concavalain-A-stimulated splenocyte staining, 2 references.<br>Anti-CD69 (mouse) - manufacturer provided PMA/ionomycin-stimulated splenocyte staining, 2 references.<br>Anti-Ly6C (mouse) - manufacturer provided mouse splenocyte staining data, 2 references.<br>Anti-Granzyme B (mouse) - manufacturer provided PMBC staining data with isotype control, 4 references.<br>Anti-phospho-Smad3 (mouse) - manufacturer provided FFPE IHC staining data with negative and positive controls, 329 references.

Eukaryotic cell lines

Policy information about cell lines

Cell line source(s)	A223 cells were derived from K15-CreΔPR1.Smad4-/-KrasG12D mice as previously described, and then passaged in syngeneic C57BL/6 mice (Jax Laboratories) twice before isolating from a tumor-draining lymph node metastasis. LY2 cells were provided by SDK. Primary mouse normal keratinocytes were generated from minced murine tongue tissue. A1419 SCC cells were derived from spontaneous 4-NQO-driven tongue carcinomas following published protocols (manuscript in preparation).
Authentication	None of the cell lines used were authenticated. We established a SNP profile for A223 cells (Dartmouse).
Mycoplasma contamination	All cell lines tested negative for mycoplasma contamination.
Commonly misidentified lines<br>(See ICLAC register)	No commonly misidentified cell lines were used in the research presented here.

Animals and other organisms

Policy information about studies involving animals; ARRIVE guidelines recommended for reporting animal research

Laboratory animals	C57BL/6 mice (Jackson Laboratories), athymic nude mice (Charles River), mTmG mice in a C57BL/6 background (ROSAmT/mG, Jackson Laboratories), and CD8-knockout mice (B6.129S2-Cd8atm1Mak/J, Jackson Laboratories) were used for all animal experiments, and all were female between 6 and 8 weeks old.
Wild animals	This study did not involve wild animals.
Field-collected samples	This study did not involve samples collected from the field.

Ethics oversight | Animal work was approved by the Institutional Animal Care and Use Committee of the University of Colorado, Anschutz Medical Campus (Aurora, CO).

Note that full information on the approval of the study protocol must also be provided in the manuscript.

Flow Cytometry

Plots

Confirm that:

☒ The axis labels state the marker and fluorochrome used (e.g. CD4-FITC).

☒ The axis scales are clearly visible. Include numbers along axes only for bottom left plot of group (a 'group' is an analysis of identical markers).

☒ All plots are contour plots with outliers or pseudocolor plots.

☒ A numerical value for number of cells or percentage (with statistics) is provided.

Methodology

Sample preparation	Tumors were excised from mice, minced, and digested with Liberase TL (Roche, 0.13 Weunsch units/ml) in GentleMACS C-tubes (Miltenyi) following the GentleMACS user protocol (Miltenyi). Digests were ground through a 100µm strainer, red blood cells lysed with eBioscience 1X RBC Lysis Buffer (Invitrogen) for 5 minutes, and finally strained through a 70µm strainer before counting and downstream analyses. 1.5 million cells from tumors in mTmG mice were stained following the BD Cytofix/Cytoperm Fixation/Permeabilization microplate staining protocol (BD Biosciences). 3 million cells from tumors in C57BL/6 mice were stained for cyTOF mass cytometry following the MaxPar Intracellular Staining Protocol and MaxPar CellID Barcoding Protocol (Fluidigm).
Instrument	Beckman Coulter Gallios 561 (A94299), BD LSRFortessa X-20 (656385), and Fluidigm Helios cyTOF system (400250 A7).
Software	Flow data was collected and analyzed by Kaluza v2.1 or CytoBank v7 (Beckman Coulter).
Cell population abundance	Cell sorting was not used in any experiments presented here.
Gating strategy	Debris was gated out of starting cell populations by excluding low FSC/SSC cells. Singlets were then confirmed using SSC/SSC area. Live cells were confirmed in in vivo experiments by Ghost Dye Violet 510 exclusion. Cells were then gated by marker expression as indicated in supplementary data. All gates were determined by fluorescence-minus-one controls, set at <0.1% positive cells for each control. For mass cytometry, live cells were confirmed by Cisplatin Cell-ID dye exclusion, followed by nucleated cell confirmation by IR-Dye Intercalator staining. CD45+ cell populations were determined by CD45 expression, and then used for subsequent analyses.

☒ Tick this box to confirm that a figure exemplifying the gating strategy is provided in the Supplementary Information.
